# Molecular Characterization and Nutritional Regulation of Two Fatty Acid Elongase (*elovl8*) Genes in Chinese Perch (*Siniperca chuatsi*)

**DOI:** 10.3390/biom15040567

**Published:** 2025-04-11

**Authors:** Yu He, Zhengyong Wen, Luo Zhou, Wanhong Zeng, Panita Prathomya, Tilin Yi, Qiong Shi

**Affiliations:** 1 School of Animal Science, Yangtze University, Jingzhou 424020, China; heyu143211@163.com (Y.H.); zhouluo3211@163.com (L.Z.); zwh15892281796@163.com (W.Z.); 2 Key Laboratory of Sichuan Province for Fishes Conservation and Utilization in the Upper Reaches of the Yangtze River, Neijiang Normal University, Neijiang 641100, China; shiqiong@szu.edu.cn; 3 College of Life Sciences, Neijiang Normal University, Neijiang 641100, China; 4 Shenzhen Key Lab of Marine Genomics, BGI Academy of Marine Sciences, BGI Marine, Shenzhen 518081, China; 5 Department of Animal and Aquatic Sciences, Faculty of Agriculture, Chiang Mai University, Chiang Mai 50200, Thailand; panita.pra@cmu.ac.th; 6 Laboratory of Aquatic Genomics, College of Life Sciences and Oceanography, Shenzhen University, Shenzhen 518057, China

**Keywords:** *Siniperca chuatsi*, *elovl8a*, *elovl8b*, nutritional regulation, LC-PUFA biosynthesis

## Abstract

Proteins for elongation of very long-chain fatty acids (ELOVLs) are critical for the synthesis of long-chain polyunsaturated fatty acids (LC-PUFAs), and they are one group of the rate-limiting enzymes responsible for the initial condensation reaction within the fatty acid elongation. Elovl8 is a newly identified member of the ELOVL protein family, and its evolutionary and functional characterizations are still rarely reported. Here, we identified two *elovl8* paralogues (named Sc*elovl8* and Sc*elovl8b*) from Chinese perch (*Siniperca chuatsi*), and then their molecular and evolutionary characteristics, as well as potential roles involved in LC-PUFA biosynthesis, were examined. The ORFs of both Sc*elovl8a* and Sc*elovl8b* genes were 810 bp and 789 bp in length, encoding proteins of 270 and 263 amino acids, respectively. Multiple protein sequence comparisons indicated that *elovl8* genes were highly conserved in teleosts, showing similar structural function domains. Meanwhile, phylogenetic analysis showed that the *elovl8* gene family was clustered into two subclades of *elovl8a* and *elovl8b*, and Sc*elovl8a* and Sce*lovl8b* shared close relationships with banded archerfish *elovl8a* and striped bass *elovl8b*, respectively. Genetic synteny and gene structure analyses further confirmed that *elovl8b* is more conserved in comparison to *elovl8a* in teleosts. In addition, Sc*elovl8a* was found to be highly expressed in the liver, while Sc*elovl8b* was most abundant in the gills. Long-term food deprivation and refeeding are verified to regulate the transcription of Sc*elovl8a* and Sc*elovl8b*, and intraperitoneal injection of fish oil (FO) and vegetable oil (VO) significantly modified their gene expression as well. In summary, our results in this study indicate that *elovl8* genes were conservatively unique to teleosts, and both *elovl8* genes might be involved in the endogenous biosynthesis of LC-PUFAs in Chinese perch. These findings not only expand our knowledge on the evolutionary and functional characteristics of both *elovl8* genes but also lay a solid basis for investigating regulatory mechanisms of LC-PUFA biosynthesis in various teleosts.

## 1. Introduction

Long-chain polyunsaturated fatty acids (LC-PUFAs) are a series of straight-chain fatty acids with three or more double bonds and containing 20 to 24 carbon atoms, which play important roles in maintaining cell membrane fluidity, regulating fat metabolism, and enhancing immunity [1,2,3,4]. LC-PUFAs, especially arachidonic acid (ARA 20:4n-6), eicosapentaenoic acid (EPA 20:5n-3), and docosahexaenoic acid (DHA 22:6n-3), are physiologically important compounds required for normal growth and development in mammals and play critical roles in reducing inflammation, cardiovascular disease, and certain types of cancers [3,5]. In teleosts, LC-PUFAs have been proved to play important roles in fish development, growth, survival, pigmentation, stress, and disease resistance [6].

LC-PUFAs are essential nutrients that can be obtained directly from exogenous food resources or synthesized from endogenous precursor substances [7]. Endogenous synthesis of LC-PUFAs requires two key rate-limiting enzymes, including fatty acyl desaturases (Fads) and fatty acid elongases (Elovls; for elongation of very long-chain fatty acids protein) [1,7]. Among them, Fads can desaturate polyunsaturated fatty acids (PUFAs) by introducing double bonds at specific positions in the carbon chain, while ELOVLs are responsible for the initial condensation reaction within the fatty acid elongation [8,9,10]. Thus far, seven members of the ELOVL protein family (termed ELOVL1~7) have been identified in various mammals based on their protein characteristics and substrate specificity [10]. Some typical features of ELOVL family members include several transmembrane α-helices (TMHs), an endoplasmic reticulum (ER) retention signal, and four ‘conserved boxes’ (KEDT-, His-, Tyr-, and Gln-boxes) involved in the catalytic site [11,12]. Among them, ELOVL1, 3, 6, and 7 mainly have the ability to prolong saturated and monounsaturated fatty acids (MUFAs), while ELOVL2, 4, and 5 mainly can prolong PUFAs [10,13,14]. Interestingly, a new *elovl* gene family member (*elovl8*) was recently discovered, and it contained two paralogs in examined teleosts [11]. Certain studies have shown that the *elovl8* gene is more closely related to *elovl4*, which could explain the previous misleading naming as *elovl4-like* [11,15].

The *elovl8b* gene was first identified in the freshwater teleost African catfish, and functional tests showed that it can prolong C18 and C20 PUFAs [16]. Subsequently, both *elovl8a* and *elovl8b* genes were identified from the herbivorous marine teleost rabbitfish (*Siganus canaliculatus*), and they were found to be widely distributed in examined tissues [11]. Interestingly, *elovl8a* expression was at the highest level in the heart, while *elovl8b* level was the highest in the brain [11]. Elovl8b was proved to have an ability to convert C18 PUFA (18:2n-6, 18:3n-3, and 18:4n-3) and C20 PUFA (20:4n-6 and 20:5n-3) to LC-PUFAs, but Elovl8a has lost this functionality [11]. Another study reported two *elovl8* genes in zebrafish (*Danio rerio*) but considered *elovl8* not unique to teleosts due to a rather unique and ample phylogenetic distribution [17]. Knocking out both *elovl8a* and *elovl8b* genes in zebrafish resulted in an accumulation of C20 fatty acids in the liver and a block in the synthesis of C22:0, as well as reduced resistance ability to cold stress [18]. The tissue distribution profile of *elovl8* in hybrid grouper (*Epinephelus fuscoguttatus* ♀× *Epinephelus lanceolatus* ♂) was similar to the pattern of *elovl8b* in rabbitfish, with higher levels in the brain and liver [11,19]. Recently, we identified a single *elovl8b* gene in yellow catfish (*Pelteobagrus fulvidraco*), while the *elovl8a* gene has been lost in the yellow catfish genome [20]. Further functional experiments revealed that diets full of LC-PUFAs can inhibit *elovl8b* transcription in the liver, suggesting the existence of a negative feedback regulation of the LC-PUFA synthetic pathway [20]. In addition, *elovl8a* and *elovl8b* genes were recently identified in an amphidromous fish (ayu sweetfish) [21]. However, the evolutionary and functional characteristics of this new elongase member are still largely unclear.

Chinese perch (*Siniperca chuatsi*), belonging to the order Centrarchiformes and the family Sinipercidae, is widely distributed in China, Russia, North Korea, South Korea, and Japan [22,23]. This fish has gained a lot of popularity due to its advantages of tender meat, tasty flavor, and absence of interstitial spines [22]. As an economically important fish in China, its annual production was approximately 400,000 tons with high commercial value [24,25]. In our present study, we identified two *elovl8* genes in Chinese perch, and then their evolutionary and functional characterizations were explored. Our findings will further enrich the understanding about endogenous biosynthetic mechanisms of LC-PUFAs and related regulatory mechanisms in teleosts.

## 2. Materials and Methods

### 2.1. Fish Sampling

Individuals of Chinese perch were purchased from a local fish farm in Neijiang City, Sichuan Province, China. They were moved into 1.5 m × 2 m × 2 m ponds at the Key Laboratory of Sichuan Province for Fishes Conservation and Utilization in the Upper Reaches of the Yangtze River, Neijiang Normal University, Neijiang, China. These fish were temporarily reared under natural light conditions (12 L/12D) for two weeks, with the water temperature between 24 and 26 °C. They were fed with larvae fish daily at 8:00 and 18:00, and the water was changed by 1/3 every 2–3 days.

Three healthy Chinese perch (average body weight: 35 ± 6.25 g) were randomly selected and anesthetized with MS-222, and then their brain, pyloric cecum, gill, gonad, heart, intestine, kidney, liver, muscle, spleen, and stomach tissues were collected. Tissue samples were immediately frozen in liquid nitrogen and then stored at −80 °C until use.

Twelve healthy Chinese perch (average body weight: 45 ± 4.38 g) were selected for a long-term starvation experiment. They were randomly divided and cultured in three tanks (n = 4 for each group) with a diameter of 1 m. One group was set as the normal feeding group (control), and the other two groups were arranged as fasting treatment and fasting-refeeding treatment, respectively. The water temperature was kept at 24–26 °C, and the dissolved oxygen was maintained at 6 mg/L or above. The experiment lasted for 18 d, and then the control group and refeeding group were fed at 18:00; at 19:00, three fish from each group were randomly selected and anesthetized with MS-222, and their livers were rapidly collected and stored as described above.

Seventy-two healthy Chinese perch (average body weight: 13 ± 1.13 g) were selected and randomly divided into three experimental groups. The control group was intraperitoneally injected with 10 μL of saline, and two experimental groups were intraperitoneally injected with 10 μL of fish oil (FO) or vegetable oil (VO), respectively. Subsequently, three fish were randomly taken from each group, anesthetized with MS-222, and their livers were rapidly collected at 0 h, 1 h, 3 h, 6 h, 12 h, 24 h, and 48 h after the injection.

In addition, 150 completed domesticated Chinese perch (average body weight: 45.5 ± 5.3 g) were purchased and randomly divided into nine 50 × 50 × 50 cm glass tanks in three treatments with three replicates per treatment. During the experiment period, the fish were fed with three different diets, including a commercial diet, a commercial diet supplemented with 2% fish oil, or a commercial diet supplemented with vegetable oil. Fatty acid composition of the experimental diets and details of the proximate composition were determined by the area normalization method, and detailed information was summarized in Table 1. At the end of the 2-week feeding trial, four fish were randomly selected from each group, and their livers were sampled and stored quickly.

### 2.2. Identification of the Scelovl8a and Scelovl8b Genes in Chinese Perch

We obtained genomic and mRNA sequences of Sc*elovl8a* and Sc*elovl8b* genes by scanning the whole-genome and transcriptome databases created by our research group previously (CNA0013732) [26], and then these isolated sequences were validated by NCBI-BLASTn with highly conserved sequence identity to corresponding genes in the NCBI database (accession numbers: XM_044218690.1 and XM_044200424.1). Quantitative primers for detecting the mRNA expression levels of both *elovl* genes in Chinese perch (listed in Table 2) were designed using Primer Premier 5.0 (Premier Biosoft International, Palo Alto, CA, USA). qRT-PCR was performed using related quantitative primers and cDNA from the liver of Chinese perch. Agarose gel electrophoresis was conducted at the end of PCR amplification to determine primer specificity and verify the existence of Sc*elovl8a* and Sc*elovl8b* transcripts in the liver.

### 2.3. Bioinformatics Analyses of the Scelovl8a and Scelovl8b Genes

Putative protein sequences were predicted using the Primer Premier 5.0 software, and then multiple protein sequence comparisons of Elovl8a and Elovl8b were performed using Clustal X (http://www.clustal.org). Transmembrane domains were identified using TMHMM Server v. 2.0 and validated by comparative analysis according to a previous study [11]. SWISS-MODEL (https://swissmodel.expasy.org) was utilized to predict the three-dimensional structures of Elovl8a and Elovl8b of several representative species. Meanwhile, we performed comparative analyses of genomic synteny and gene structure of both *elovl8a* and *elovl8b* genes by using some representative teleost genomic data and/or related results from previous reports to determine their conservation during teleost evolution [11,20].

In addition, a phylogenetic analysis was conducted to explore the evolutionary history of the *elovl8* gene family. Protein sequences used for the phylogeny were downloaded from the NCBI or Ensembl database. After calculation, JTT + G was chosen as the best model to construct a phylogenetic tree using the neighbor-joining (NJ) method. Meanwhile, both Bayesian inference and the maximum likelihood methods were performed, sharing a similar topology to validate the phylogeny (see more details in Supplementary Figures S1 and S2). Finally, the accuracy of the tree topology was assessed by a non-parametric bootstrap analysis with 1000 resampling replicates. Resources of the selected ELOVL family proteins are provided in Table 3.

### 2.4. Total RNA Extraction and cDNA Preparation

Total RNA was extracted from 10 to 20 mg of each sample using the TRIzol agent (Thermo Fisher Scientific, Waltham, MA, USA), and then its concentration was measured by a micro-protein nucleic acid meter (Thermo Fisher Scientific) for maintenance at 100–1000 ng/μL. Subsequently, the quality of extracted RNA was examined by gel electrophoresis [27,28]. Finally, the extracted RNA was stored at −80 °C for further utilization.

In general, 1 μg of total RNA was reverse transcribed into cDNA using a cDNA. First Strand Synthesis kit (Novoprotein, Shanghai, China). The final volume of the reaction was set as 20 μL, and the reaction procedure was designed as follows: 50 °C for 15 min and then 85 °C for 30 s. After that, the quality of transcribed cDNA was measured by PCR and gel electrophoresis, and the *β-actin* gene was used as the internal reference.

### 2.5. Quantitative Real-Time PCR

Quantitative real-time PCR (qRT-PCR) was conducted to detect the transcription levels of Sc*elovl8a* and Sc*elovl8b* on a Light Cycler real-time system (Roche Diagnostics, Indianapolis, IN, USA) with a final volume of 20 μL. Meanwhile, the relative expression levels of mRNAs were normalized by *β-actin* after assessing the stability of five internal reference genes. The primers of *β-actin* (Table 1) were obtained and selected from a previous study [29]. Finally, relative expression levels were calculated with the Pfaffl method [30]. Specific primer sequences used for the qRT-PCRs are listed in Table 1.

### 2.6. Statistical Analysis

SPSS 26.0 (IBM, Armonk, NY, USA) and GraphPad Prism 5.0 (GraphPad Prism Software Inc., San Diego, CA, USA) were applied for statistical analysis. All data were presented as mean ± standard error of the mean (SEM). Significant differences were determined using one-way analysis of variance (ANOVA), followed by Tukey’s test, and differences were designed as significant when *p* < 0.05.

## 3. Results

### 3.1. Presence of Both elovl8a and elovl8b Genes in Chinese Perch

**Figure 1:**
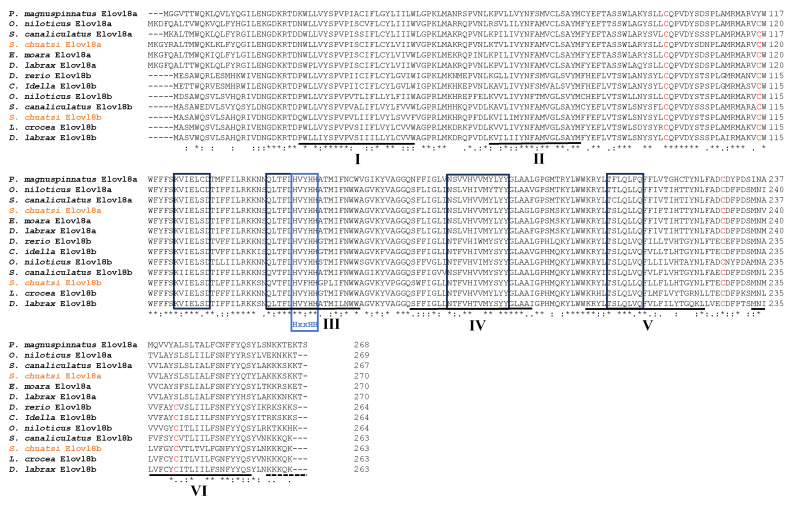
Multiple protein sequence alignment of Elovl8a and Elovl8b among various fishes. Names of the Chinese perch Elovl8a and Elovl8b are highlighted in orange. Cysteines are marked in red. Six conserved transmembrane α-helix domains are labeled (I to VI). Black boxes show the four conserved elongase motifs. Blue boxes show the histidine structural domain (HXXHH). Underlined dotted lines represent the putative endoplasmic reticulum (ER). Asterisks (*) indicate identical residues (fully conserved residues); colons (:) and periods (.) indicate highly conserved and semi-conserved amino acids, respectively.

In our present study, both *elovl8a* and *elovl8b* genes were identified for the first time from Chinese perch. The open reading frame (ORF) of Sc*elovl8a* was 810 bp, encoding a protein of 270 amino acid (aa) residues, whereas the ORF of Sc*elovl8b* was 789 bp, encoding a protein of 263 aa (Figure 1). In comparison with several teleost Elovl8s, the protein sequences of ScElovl8s were found to be highly conserved, containing six conserved transmembrane α-helical structural domains (TMHs), four conserved functional elongase motifs, a highly conserved histidine structural domain, and three highly conserved cysteine residues (see more details in Figure 1). Notably, Elovl8a is commonly 5 aa longer than Elovl8b in teleosts. Additionally, a highly conserved cysteine residue was identified in the sixth transmembrane region of Elovl8b rather than Elovl8a (see Figure 1).

### 3.2. Predicted 3D Structures of Both Elovl8a and Elovl8b in Chinese Perch

**Figure 2:**
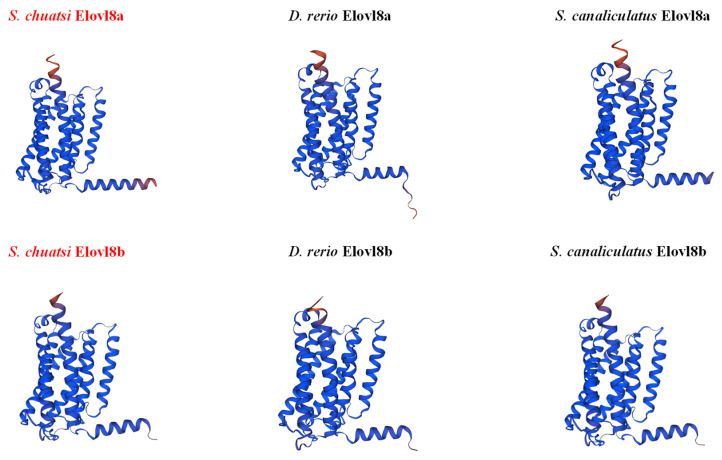
Prediction of the 3D structures of Elovl8a and Elovl8b. Chinese perch Elovl8a and Elovl8b are highlighted in red.

The 3D structures of Elovl8 proteins of several representative fish species were predicted, and our results showed that both ScElovl8a and ScElovl8b were highly similar to those in yellow catfish and rabbitfish (Figure 2). These Elovl8s have eight longer α-helix structures, seven of which are similar in positional space and size, with a relatively less coherent α-helix structure at the N-terminus (see more details in Figure 2). It is evident that the Elovl8b structures of the three species are more conserved in comparison with Elovl8a.

### 3.3. Phylogenetic Tree of the Examined Elovl Genes in Teleost

**Figure 3:**
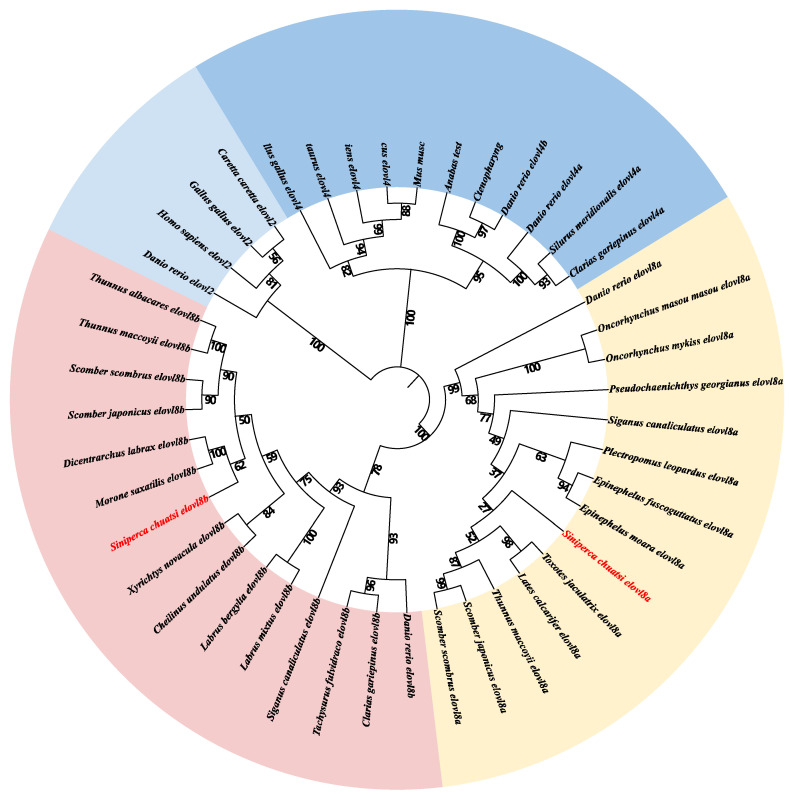
Evolutionary relationship among *elovl2*, *elovl4*, and *elovl8* in representative vertebrates. It was constructed with the MEGA-X software based on public protein datasets (Table 3). Values at the nodes represent bootstrap percentages from 1000 replicates. The Chinese perch *elovl8a* and *elovl8b* were marked in red. The tetrapod *elovl2* family is set as the outgroup.

For a better understanding of the evolutionary relationship of *elovl*s in teleosts, we constructed a relative phylogenetic tree with MEGA-X using the protein sequence datasets of representative teleost species. The tree is obviously divided into three subfamilies, including *elovl2*, *elovl4*, and *elovl8*; among them, the *elovl4* and *elovl8* clades are clustered together (Figure 3). Meanwhile, the *elovl8* clusters were subdivided into *elovl8a* and *elovl8b* branches, and *elovl8a* and *elovl8b* of the Chinese perch were closely related to corresponding paralogs in the Percomorpha (see more details in Figure 3).

### 3.4. Gene Structures and Genomic Synteny of Both elovl8 Genes in Teleost

**Figure 4:**
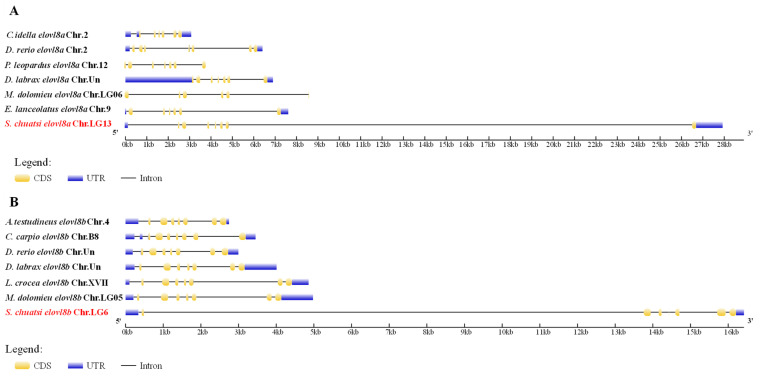
A comparative analysis of the gene structures of *elovl8a* (**A**) and *elovl8b* (**B**) genes among different fish species. The colorful boxes and lines represent the exons and introns, respectively. Blue and yellow boxes mark the UTR and CDS, respectively. Names of the Chinese perch *elovl8a* and *elovl8b* genes are highlighted in red.

A comparative analysis of gene structures can reveal their variability across various species. In this study, the *elovl8a* gene contains eight exons and seven introns in Chinese perch and zebrafish (*Danio rerio*), whereas the *elovl8a* gene of grass carp (*Ctenopharyngodon idella*), smallmouth bass (*Micropterus dolomieu*), leopard coralgrouper (*Plectropomus leopardus*), and giant grouper (*Epinephelus lanceolatus*) contains seven exons and six introns, but the European seabass (*Dicentrarchus labrax*) *elovl8a* gene only has six exons and five introns (Figure 4A). Differently, the structure of the *elovl8b* gene in Chinese perch was similar to that of those in climbing perch (*Anabas testudineus*), zebrafish, European seabass, greater amberjack (*Seriola dumerili*), and smallmouth bass; all of them have eight exons and seven introns (Figure 4B). Interestingly, the gene structures of *elovl8a* and *elovl8b* genes are different in smallmouth bass and European seabass, whereas they are consistent in Chinese perch (Figure 4).

**Figure 5:**
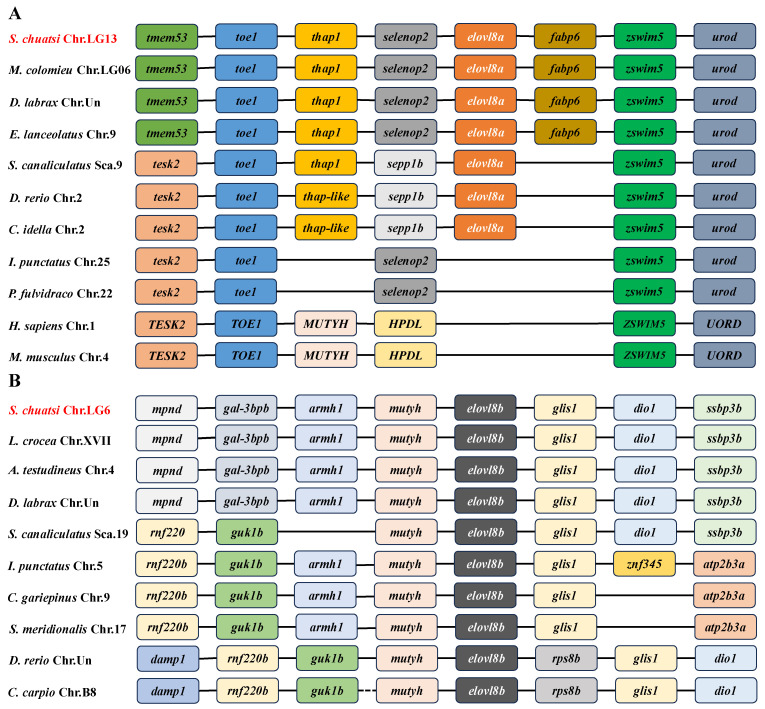
Genomic synteny of the *elovl8a* (**A**) and *elovl8b* (**B**) genes in representative vertebrates. Target names are highlighted in red. Each same colorful block represents the same gene. Solid lines indicate the absence of corresponding gene(s) in the intergenic region.

In addition, comparative genomic synteny was obtained to investigate the evolutionary process of *elovl8* genes in vertebrates. As detailed in Figure 5, the *elovl8a* is absent in the genome of mammals and Siluriformes fishes, whereas both *elovl8a* and *elovl8b* genes are present in Chinese perch, rabbitfish, European perch, and zebrafish. The genetic loci of *elovl8a* and *elovl8b* genes are significantly variable; however, two relatively conserved gene clusters, *toe1-thap1-elovl8a-zswim5-urod* and *mutyh-elovl8b-glis1*, are identified in the genome of these representative species.

### 3.5. Tissue Distribution Pattern of Both elovl8a and elovl8b Genes in Chinese Perch

**Figure 6:**
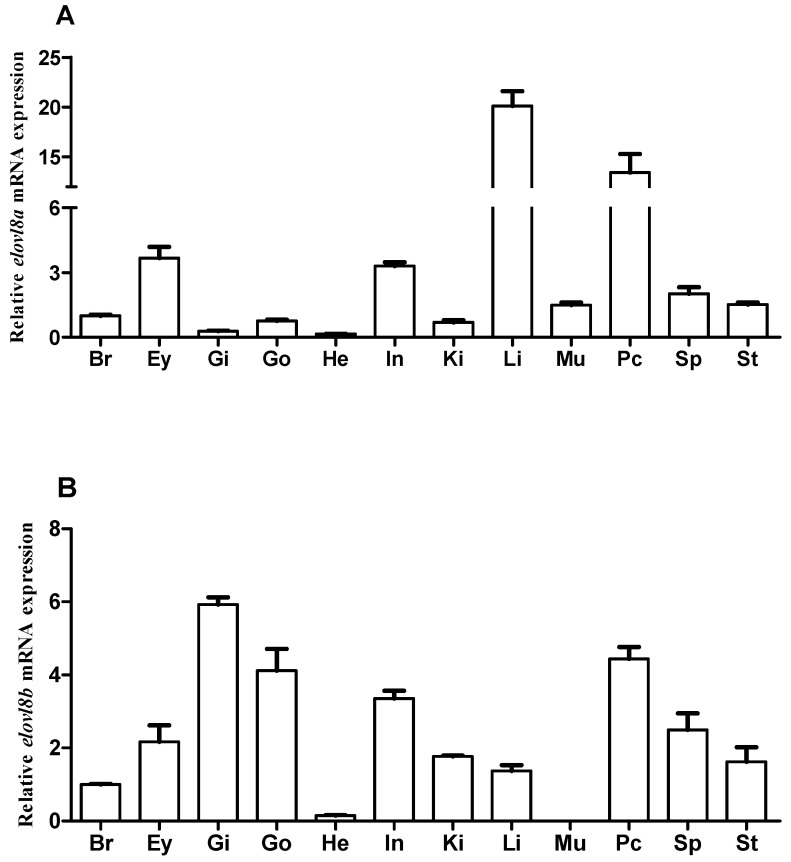
Relative mRNA expression levels of *elovl8a* (**A**) and *elovl8b* (**B**) in different tissues of Chinese perch. Abbreviations: brain (Br); eye (Ey); gill (Gi); gonad (Go); heart (He); intestine (In); kidney (Ki); liver (Li); muscle (Mu); pyloric cecum (Pc); spleen (Sp); stomach (St). Data are presented as means ± SEM (n = 3).

Distribution patterns of both *elovl8a* and *elovl8b* genes were determined by qRT-PCR, and a total of 11 tissues (including brain, pyloric cecum, gill, gonad, heart, intestine, kidney, liver, muscle, spleen, and stomach) were detected in Chinese perch. Obviously, the *elovl8a* gene was extensively detectable in all measured tissues, with relatively higher expression levels in the liver and pyloric cecum (Figure 6A). Similarly, the *elovl8b* gene was also widely expressed in these measured tissues, with the highest expression level in gills, although no expression level was detected in the muscle (Figure 6B).

### 3.6. Effects of Different Nutritional Status on Transcriptional Changes of elovl8a and elovl8b Genes in Chinese Perch

**Figure 7:**
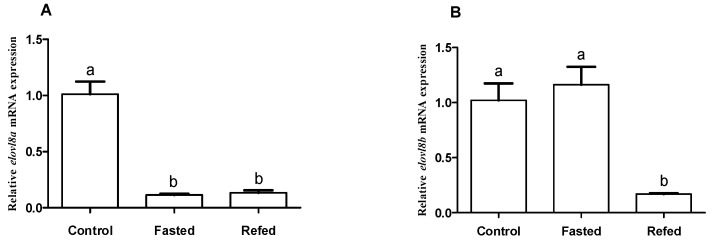
Effects of long-term (18 d) fasting and refeeding on transcription of liver *elovl8a* (**A**) and *elovl8b* (**B**) in Chinese perch. Data are presented as mean ± SEM (n = 3). A significant difference (*p* < 0.05) between groups was determined using one-way ANOVA. Groups with significant differences are labeled with different letters above corresponding bars.

In the long-term (18 d) food deprivation experiment of Chinese perch, we explored transcriptional changes of both *elovl8a* and *elovl8b* genes in the liver using qRT-PCR and also examined the expression pattern of both genes under different nutritional statuses. As shown in Figure 7, the transcription of *elovl8a* in Chinese perch was significantly decreased during the long-term starvation, while there was no significant change after refeeding compared with the starvation group (Figure 7A). In contrast, the expression of *elovl8b* did not change significantly during the long-term starvation compared with the control group, while it decreased significantly after refeeding compared with the control and starvation groups (Figure 7B).

### 3.7. Effects of Fish Oil and Vegetable Oil Administration on the Expression Levels of Both elovl8a and elovl8b Genes in Chinese Perch

**Figure 8:**
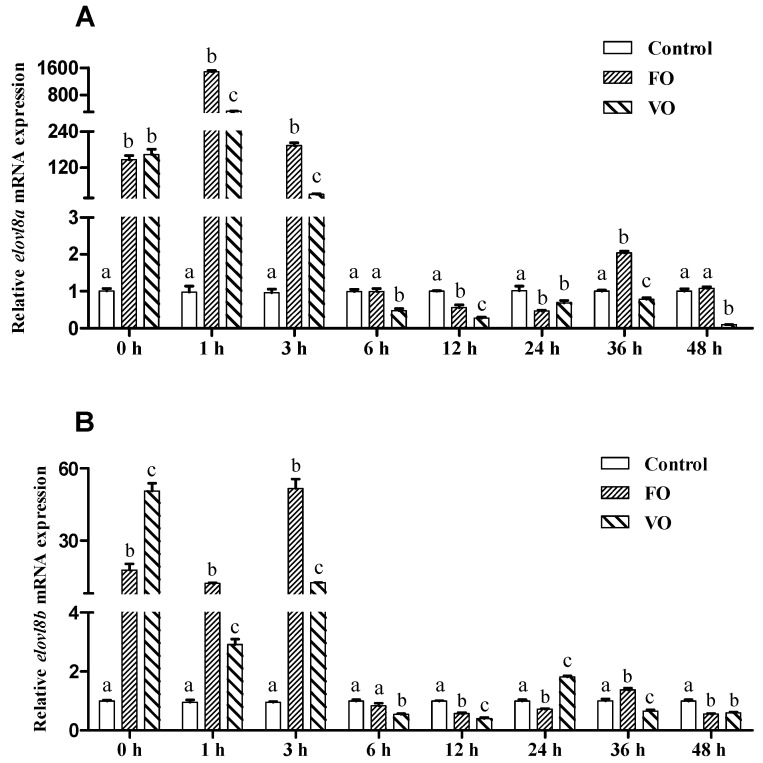
Effects of fish oil injection on *elovl8a* (**A**) and *elovl8b* (**B**) transcription in the liver of Chinese perch. *β-actin* was used as the internal reference gene. Data are expressed as mean ± standard error (n = 3). A significant difference (*p* < 0.05) between groups was determined using one-way ANOVA (with different labels above corresponding bars).

Transcriptional changes of both *elovl8a* and *elovl8b* in response to intraperitoneal injection of fish oil (FO) and vegetable oil (VO) were detected. Our results showed that the transcription of both *elovl8a* and *elovl8b* was significantly increased at 0, 1, and 3 h after intraperitoneal injection of FO and VO in Chinese perch and then decreased at 6~48 h after injection with some variations (Figure 8). Interestingly, at 1, 3, 6, 12, 36, and 48 h after injection, transcription of *elovl8a* in the FO group was significantly higher than in the VO group (Figure 8A), and a similar pattern was observed for *elovl8b* at 1, 3, 6, 12, and 36 h after administration (Figure 8B).

### 3.8. Effects of Dietary Fish Oil and Vegetable Oil Supplementation on Both elovl8a and elovl8b Transcriptions in Chinese Perch

**Figure 9:**
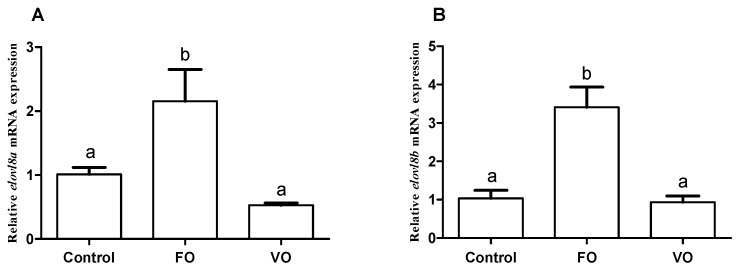
Effects of different diets on *elovl8a* (**A**) and *elovl8b* (**B**) transcription in the liver of Chinese perch. *β-actin* was used as the internal reference gene. Data are expressed as mean ± standard error (n = 3). A significant difference (*p* < 0.05) between groups was determined using one-way ANOVA (with different labels above corresponding bars).

To investigate the transcriptional change patterns of both *elovl8a* and *elovl8b* genes in response to different dietary fish oil and vegetable oil supplementations, we detected their mRNA expression levels in the liver of Chinese perch. Our results showed that the hepatic *elovl8a* transcription level was significantly higher in fish fed with 2% FO supplementation diet than that of those fed with the control and with 2% VO supplementation diets (Figure 9A). A consistently similar pattern was observed for the *elovl8b* gene in Chinese perch (Figure 9B).

## 4. Discussion

LC-PUFAs are essential nutrients for several important physiological processes, including maintenance of cell membrane structure, energy metabolism, gene regulation, cell signaling, and immune function [31,32]. As a key rate-limiting enzyme in the endogenous biosynthesis of LC-PUFAs, ELOVL can extend the carbon chain of LC-PUFAs [10,20]. Several members of the ELOVL family (ELOVL1~7) have been extensively studied, while less is known about the molecular and functional characteristics of the new member ELOVL8 [10,17,33]. In our present study, two elongase genes (named as *elovl8a* and *elovl8b*) of Chinese perch were identified, and their ORFs were 810 and 789 bp, encoding 270 and 263 aa, respectively. The ORF length of Chinese perch *elovl8a* is different from that in rabbitfish [11], implying that *elovl8a* may be variable in teleosts. In contrast, the ORF of *elovl8b* showed high consistency among various teleosts, encoding approximately 263 aa, such as in rabbitfish [11], hybrid grouper [19], and yellow catfish [20]. These findings suggest that *elovl8b* is more conserved in comparison with *elovl8a* in teleosts.

Multiple protein sequence alignments revealed that both Elovl8a and Elovl8b of Chinese perch possess some ELOVL family features (Figure 1), including four conserved regions that are related to catalysis, six predicted a-transmembrane regions, a histidine box (HXXHH), and an endoplasmic reticulum (ER) retention signal. These data are consistent with several previous reports [11,15,19,20], indicating that Elovl8 proteins may be able to participate in LC-PUFA biosynthesis in teleosts. Subsequently, similar 3D structures of Elovl8 proteins were observed in several representative fish species (Figure 2), suggesting that both Elovl8a and Elovl8b are highly conserved with potentially similar functions [33].

Our phylogenetic analysis showed that the *elovl8* gene family is closely related to but distinctly different from the *elovl4* gene family (Figure 3), which is consistent with previous studies in yellow catfish, rabbitfish, ayu sweetfish, and threadfin fish [11,20,21,33]. Among them, *elovl8* can be further divided into *elovl8a* and *elovl8b* subclades, and the Chinese perch *elovl8a* and *elovl8b* shared close relationships with banded archerfish *elovl8a* and striped bass *elovl8b*, respectively. Our findings are in line with those reports in previous studies [11,20], implying that two paralogs may have commonly existed in teleosts, and this phenomenon may be generated by the teleost-specific whole genome duplication event [11]. Meanwhile, both *elovl8a* and *elovl8b* of Chinese perch originated after zebrafish *elovl8*s, indicating that the evolutionary process of *elovl8* genes is consistent with species evolution [27,33,34,35]. However, *elovl8a* has been lost in some fish species, such as yellow catfish, African catfish, channel catfish, and threadfin fish (*Eleutheronema rhadinum*); this phenomenon is probably due to functional redundancy between *elovl8a* and *elovl8b* isotypes early in evolution [16,20,33]. Meanwhile, the ability of Elovl8 to prolong LC-PUFAs was first reported in African catfish by using a yeast heterologous expression assay [16]. Consistently, a previous study [11] has shown that rabbitfish Elovl8b possesses an ability to extend C18 (18:2n- 6,18:3 n-3, and 18:4n-3) and C20 (20:4n-6 and 20:5n-3) PUFAs to long-chain fatty acids, whereas Elovl8a is not involved in such LC-PUFA synthesis. However, several recent studies reported that both Elovl8a and Elovl8b may play a potential role in fatty acid biosynthesis in zebrafish and ayu sweetfish [17,18]. In addition, previous studies suggested that *elovl8* may be only found in teleosts, but recent reports revealed that *elovl8* may also be present in some amphibians, reptiles, and other chordates as well [11,17]. Interestingly, a recent study [34] stated that *elovl8-like* genes were identified in echinoderms, implying a complex evolutionary history of this gene family in various animals.

Gene structure comparison revealed that the number of introns and exons of *elovl8a* genes are variable (Figure 4A), while the structure of *elovl8b* genes was more conserved (both existed in seven coding regions; Figure 4B), which is consistent with those findings in rabbitfish and yellow catfish [11,20]. These results suggest that Elovl8b may play more important roles than its paralog Elovl8a. Meanwhile, genomic synteny further indicated that both *elovl8a* and *elovl8b* genes are conserved in teleosts (Figure 5). Interestingly, the *TESK2-TOE1* and *ZSWIM5-UROD* flanking genes were found in mammalian humans and mice, and both gene clusters are associated with the *elovl8a* gene; the flanking of the teleost *elovl8b* gene was also present, indicating that the *elovl8* may have ever extensively existed in vertebrates, and two isoforms in teleosts may be due to the teleost-specific genome duplication event [20,36,37].

Tissue distribution patterns showed that both *elovl8a* and *elovl8b* genes were expressed in almost all examined tissues in Chinese perch (Figure 6). The *elovl8a* was mainly distributed in the liver, pyloric cecum, eye, and intestine (Figure 6A), whereas *elovl8b* was relatively high in the gill and pyloric cecum (Figure 6B), suggesting that the *elovl8a* and *elovl8b* genes in Chinese perch may play different physiological roles in the same tissues. These distribution patterns of Chinese perch, however, are different from those in rabbitfish and yellow catfish [11,20], implying that the distribution patterns of *elovl8* genes may be species-specific, which could be caused by various inhabiting environments. These findings indicate that both *elvol8a* and *elovl8b* are involved in crucial physiological processes in various tissues, while functional differentiation may also exist between the two paralogs. Notably, both *elovl8a* and *elovl8b* have high expression in the liver, which may be due to the fact that the liver is the main organ for lipid metabolism and synthesis [11,20,33,38,39,40].

Previous studies have shown that different levels of ingested nutrients or different nutritional statuses can affect the biosynthesis of LC-PUFAs [39,41,42,43]. In Senegalese sole, refeeding significantly decreased the expression of hepatic Δ4*fad* and *elovl5* genes after long-term starvation [40]. In yellow catfish, however, long-term starvation caused a significant decrease in hepatic *fad6* gene expression with no significant change after refeeding [44]. In our present study, the Chinese perch hepatic *elovl8a* expression was significantly decreased after long-term starvation, with no significant change in expression after refeeding either (Figure 7A), indicating that long-term food deprivation may restrict the process of HUFA biosynthesis, but rescue with food supplements cannot obviously return the HUFA biosynthesis after this long-term food deprivation [44]. Differently, long-term fasting did not alter the haptic *elovl8b* expression, but refeeding significantly decreased its expression after refeeding (Figure 7B), implying that *elovl8b* may play important roles in maintaining normal physiological activity during starvation, and this is functionally different from *elovl8a* in Chinese perch.

Meanwhile, intraperitoneal injection experiments were conducted to further explore the functional traits of Elovl8s in Chinese perch. Our results showed that the expression levels of hepatic *elovl8a* and *elovl8b* genes were significantly increased at 0, 1, and 3 h after administration with FO and VO (Figure 8), suggesting that both FO and VO administration can stimulate the LC-PUFAs biosynthetic pathways in Chinese perch and thus compensate for the lower levels of essential LC-PUFAs in the commercial diet [20]. Besides, the expression pattern of the *elovl8a* gene (Figure 8A) was similar to that of the *elovl8b* gene (Figure 8B), implying that the *elovl8a* gene is also involved in the LC-PUFAs biosynthesis pathway in Chinese perch [17]. Notably, loss of the *elovl8a* gene in some teleost genomes is probably due to functional redundancy between *elovl8a* and *elovl8b* isotypes, such as in yellow catfish, African catfish, channel catfish, and threadfin fish [11,16,20,33]. Generally speaking, the variations in *elovl8* in various teleosts may be due to the loss of genes or functions resulting from the overlapping of LC-PUFAs biosynthesis pathways in vivo as a result of the combined effects of different nutritional levels, feeding habits, and ecological habits [5,20,45].

Further in vivo experiments showed that the expression profiles of the Chinese perch hepatic *elovl8a* and *elovl8b* genes were regulated by dietary FO or VO supplementation (Figure 9), which is similar to those reports in rabbitfish and yellow catfish [11,20]. In our present study, dietary FO supplementation significantly improved the expression levels of both hepatic *elovl8a* and *elovl8b* genes, whereas dietary VO supplementation had no effect in Chinese perch (see Figure 9A,B). These findings are consistent with previous studies, which have shown that the *elovl8* genes prefer to extend n-3 LC-PUFA substrates rather than n-6 LC-PUFA substrates in rabbitfish and African catfish [11,16]. In our current study, the FO diet contained a higher content of n-3 LC-PUFA substrates and thus significantly induced the transcription of *elovl8* genes, suggesting that both *elovl8* genes may have the ability to prolong LC-PUFAs in Chinese perch. Similar results were observed in zebrafish, since knockdown of its *elovl8a* and *elovl8b* genes resulted in accumulation of C20 LC-PUFAs in vivo and impaired synthesis of C22 LC-PUFAs [18].

## 5. Conclusions

In summary, two fatty acid elongase genes (*elovl8a* and *elovl8b*) were identified from Chinese perch in our present study. Bioinformatics analyses revealed that both elovl8 genes were highly conserved, implying their evolutionary and functional conservation in Chinese perch. Species-specific tissue distribution patterns suggest functional diversity of *elovl8* genes in various teleosts. Meanwhile, different nutritional statuses and dietary fatty acid supplementation can affect the transcription of both *elovl8* genes and thus regulate the LC-PUFA biosynthesis in Chinese perch. All in all, our findings provide novel insights into the evolutionary and functional properties of *elovl8* genes in teleosts, as well as lay a solid foundation for a better understanding of the regulatory mechanisms of LC-PUFAs in vertebrates.

## Figures and Tables

**Table 1:** Detailed fatty acids and proximate composition in the experimental diets.

Category	Experimental Diets		
	Control	2% FO	2% VO
Proximate composition (%)			
Moisture	7.30	7.70	7.30
Crude protein	50.83	49.23	49.13
Crude lipid	12.20	13.70	13.60
Ash	13.40	13.40	13.00
Fatty acids (%Crude lipid)			
C12:0	0.046	0.044	0.040
C13:0	0.018	0.024	0.016
C14:0	2.00	2.18	1.60
C15:0	0.26	0.21	0.15
C16:0	16.20	16.50	15.80
C17:0	0.68	0.69	0.50
C18:0	5.05	5.18	4.70
C20:0	0.40	0.49	0.41
C21:0	0.056	0.071	0.049
C23:0	0.076	0.068	0.0644
C24:0	0.33	0.31	0.31
Total SFA	25.11	25.76	23.64
C14:1n-5	0.025	0.026	0.028
C16:1n-7	2.21	2.80	1.91
C17:1n-7	0.31	0.4	0.277
C18:1n-9	21.2	21.4	22.5
C20:1n-11	0.71	0.71	0.59
C22:1n-9	0.12	0.14	0.12
Total MUFA	24.58	25.51	25.42
C18:2n-6	33.80	32.20	37.50
C20:3n-6	0.072	0.10	0.067
C20:4n-6	0.57	0.70	0.51
C22:2n-6	0.017	0.012	0.011
Total n-6 PUFA	34.45	33.02	38.08
C18:3n-3	4.56	4.20	3.84
C20:3n-3	0.055	0.056	0.053
C20:5n-3	4.35	4.52	3.41
C22:6n-3	6.72	6.79	5.43
Total n-3 PUFA	15.68	15.57	12.73
Fatty acids (%Experimental diets)			
C18:2n-6	4.12	4.52	5.10
C18:3n-3	0.55	0.58	0.52
C20:5n-3	0.53	0.62	0.46
C22:6n-3	0.82	0.93	0.74

**Table 2:** Primer sequences for the qRT-PCR studies.

Primer Name	Primer Sequence (5′-3′)
*elovl8a*-qF	ATTGGCTGCTGGTCTACTC
*elovl8a*-qR	CAGGAAGAGGCTGTGAACT
*elovl8b*-qF	GGCGGGAGTCAAGTATGT
*elovl8b*-qR	TAGGGACGTGAGGTATCG
*β-actin*-qF	GTGCGTGACATCAAGGAGAAG
*β-actin*-qR	GGAAGGAAGGCTGGAAGAGG

**Table 3:** Resources of the examined ELOVL sequences in Ensembl or GenBank.

Number	Species	Protein	Protein ID
1	*Scomber scombrus*	Elovl8a	XP_062285127.1
2	*Scomber japonicus*	Elovl8a	XP_053185701.1
3	*Thunnus maccoyii*	Elovl8a	XP_042283429.1
4	*Lates calcarifer*	Elovl8a	XP_018531677.1
5	*Toxotes jaculatrix*	Elovl8a	XP_040911096.1
6	*Plectropomus leopardus*	Elovl8a	XP_042354264.1
7	*Epinephelus moara*	Elovl8a	XP_049888834.1
8	*Epinephelus fuscoguttatus*	Elovl8a	XP_049453958.1
9	*Pseudochaenichthys georgianus*	Elovl8a	KAA0719250.1
10	*Danio rerio*	Elovl8a	NP_001070061.1
11	*Oncorhynchus mykiss*	Elovl8a	XP_021466941.1
12	*Oncorhynchus masou masou*	Elovl8a	XP_064784052.1
13	*Siganus canaliculatus*	Elovl8a	QMU95576.1
14	*Clarias gariepinus*	Elovl8b	XP_053360794.1
15	*Cheilinus undulatus*	Elovl8b	XP_041647543.1
16	*Danio rerio*	Elovl8b	NP_001191453.1
17	*Dicentrarchus labrax*	Elovl8b	XP_051234026.1
18	*Morone saxatilis*	Elovl8b	XP_035509917.1
19	*Labrus bergylta*	Elovl8b	XP_052466620.1
20	*Labrus mixtus*	Elovl8b	XP_060889255.1
21	*Scomber japonicus*	Elovl8b	XP_053178828.1
22	*Scomber scombrus*	Elovl8b	XP_062279998.1
23	*Siganus canaliculatus*	Elovl8b	QMU95577.1
24	*Tachysurus fulvidraco*	Elovl8b	XP_047655843.1
25	*Thunnus albacares*	Elovl8b	XP_044218547.1
26	*Thunnus maccoyii*	Elovl8b	XP_042273790.1
27	*Xyrichtys novacula*	Elovl8b	CAJ1060369.1
28	*Danio rerio*	Elovl2	NP_001035452.1
29	*Homo sapiens*	ELOVL2	NP_060240.3
30	*Caretta caretta*	Elovl2	XP_048695790.1
31	*Gallus gallus 2*	ELOVL2	NP_001184237.1
32	*Clarias gariepinus*	Elovl4a	XP_053351126.1
33	*Silurus meridionalis*	Elovl4a	XP_046702911.1
34	*Danio rerio*	Elovl4a	NP_957090.1
35	*Ctenopharyngodon idella*	Elovl4b	XP_051736330.1
36	*Anabas testudineus*	Elovl4b	XP_026196457.1
37	*Danio rerio*	Elovl4b	NP_956266.1
38	*Bos taurus*	ELOVL4	NP_001092520.1
40	*Gallus gallus*	ELOVL4	XP_046794870.1
41	*Homo sapiens*	ELOVL4	NP_073563.1
42	*Mus musculus*	ELOVL4	NP_683743.2
43	*Rattus norvegicus*	ELOVL4	NP_001178725.1

## Data Availability

The raw data supporting the conclusions of this article will be made available by the authors upon request without undue reservation.

## Supplementary Materials

The following supporting information can be downloaded at: https://www.mdpi.com/article/10.3390/biom15040567/s1, Figure S1: Evolutionary relationship among *elovl2*, *elovl4*, and *elovl8* in representative vertebrates inferred by maximum likelihood method. Values at the nodes represent bootstrap percentages from 1000 replicates. The Chinese perch *elovl8a* and *elovl8b* were marked in red. Tetrapod *elovl2* family is set as the outgroup. Figure S2: Evolutionary relationship among *elovl2*, *elovl4*, and *elovl8* in representative vertebrates constructed with Bayesian inference method. The posterior probability is shown on the branch. The Chinese perch *elovl8a* and *elovl8b* were marked in red. Tetrapod *elovl2* family is set as the outgroup.
