# Supplementary figures and images for: Molecular Characterization and Nutritional Regulation of Two Fatty Acid Elongase (elovl8) Genes in Chinese Perch (Siniperca chuatsi)
Source: Biomolecules. 2025 Apr 11;15(4):567. doi: 10.3390/biom15040567 (PMC12025145; doi:10.3390/biom15040567)

Supplementary figure S1 (ML tree)

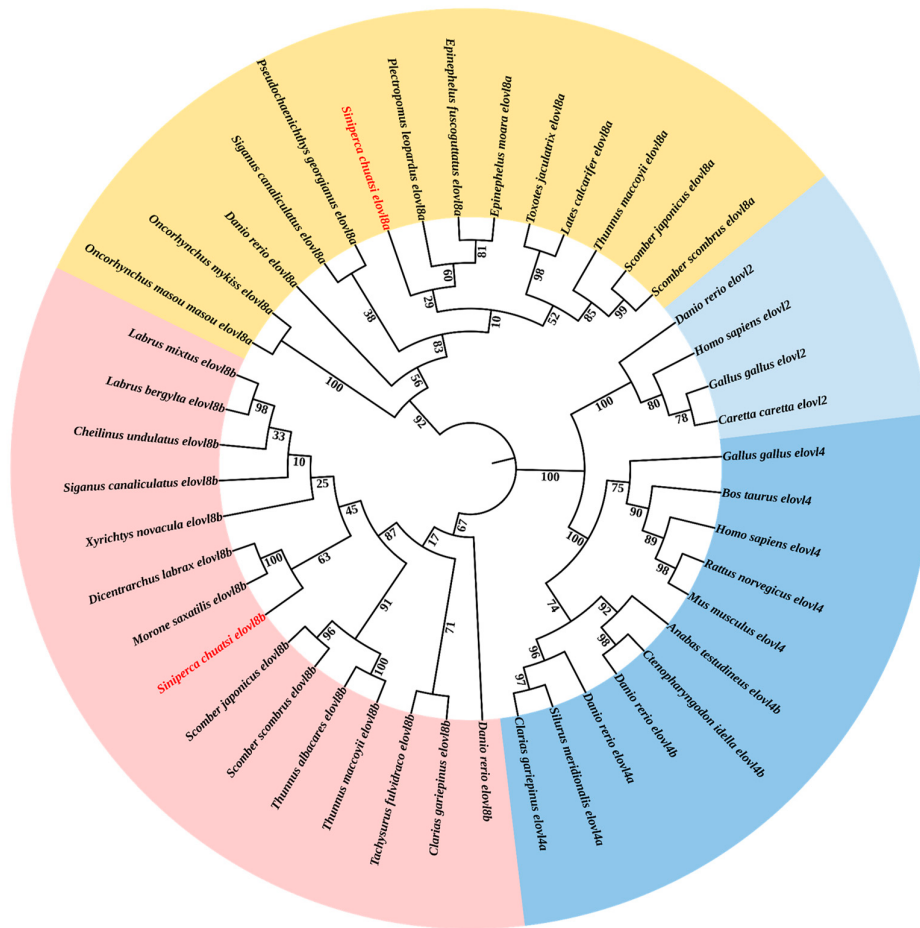

Supplementary figure S2 (BI tree)

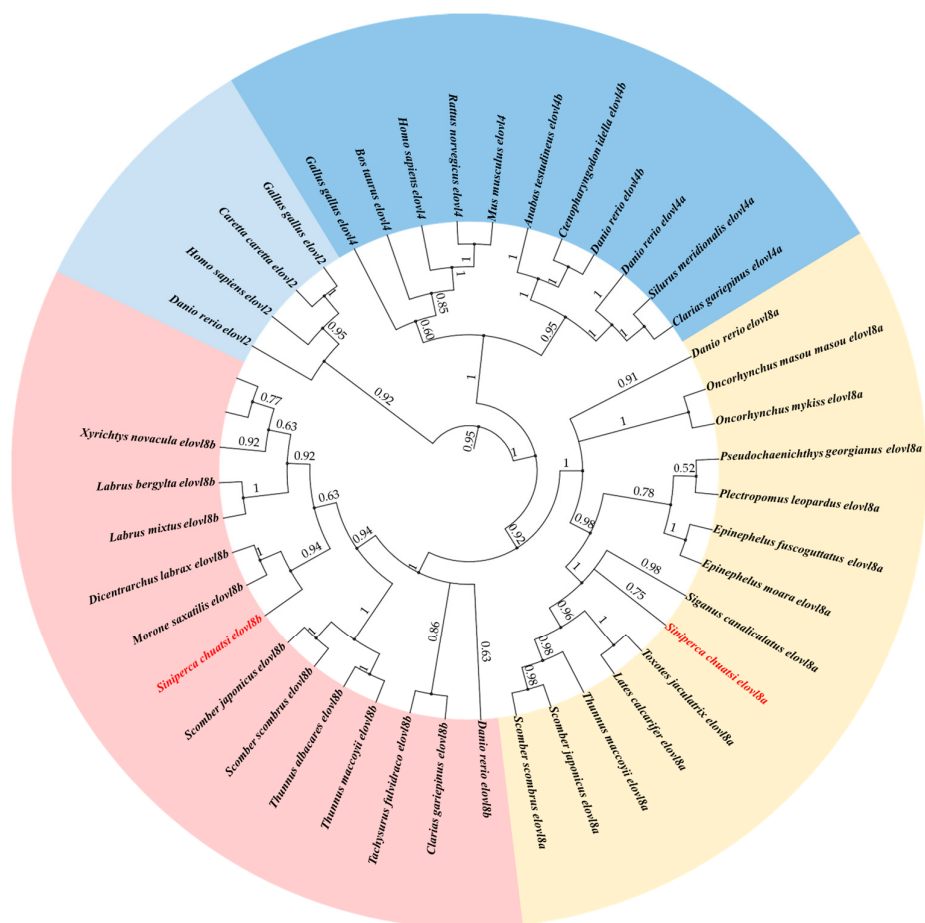

Supplement: Supplementary file 1 [file biomolecules-15-00567-s001.zip › biomolecules-3507276-supplementary.pdf]
